# A High-Performance Self-Supporting Electrochemical Biosensor to Detect Aflatoxin B1

**DOI:** 10.3390/bios12100897

**Published:** 2022-10-20

**Authors:** Yunfei Zhang, Tingting Lin, Yi Shen, Hongying Li

**Affiliations:** 1 School of Food Science and Engineering, South China University of Technology, Guangzhou 510640, China; 2 Overseas Expertise Introduction Center for Discipline Innovation of Food Nutrition and Human Health (111 Center), Guangzhou 510640, China; 3 Sino-Singapore International Joint Research Institute, Guangzhou Knowledge City, Guangzhou 510663, China; 4 Institute of High-Performance Computing, Agency for Science, Technology and Research, Singapore 138632, Singapore

**Keywords:** self-supporting electrodes, electrochemical biosensors, aflatoxin B1, horseradish peroxidase, hydrogen peroxide

## Abstract

High-performance electrochemical biosensors for the rapid detection of aflatoxin B1 (AFB_1_) are urgently required in the food industry. Herein, a multi-scaled electrochemical biosensor was fabricated by assembling carboxylated polystyrene nanospheres, an aptamer and horseradish peroxidase into a free-standing carbon nanofiber/carbon felt support. The resulting electrochemical biosensor possessed an exceptional performance, owing to the unique structures as well as the synergistic effects of the components. The 3D porous carbon nanofiber/carbon felt support served as an ideal substrate, owing to the excellent conductivity and facile diffusion of the reactants. The integration of carboxylated polystyrene nanospheres with horseradish peroxidase was employed as a signal amplification probe to enhance the electrochemical responses via catalyzing the decomposition of hydrogen peroxide. With the aid of the aptamer, the prepared sensors could quantitatively detect AFB_1_ in wine and soy sauce samples via differential pulse voltammetry. The recovery rates of AFB_1_ in the samples were between 87.53% and 106.71%. The limit of detection of the biosensors was 0.016 pg mL^−1^. The electrochemical biosensors also had excellent sensitivity, reproducibility, specificity and stability. The synthetic strategy reported in this work could pave a new route to fabricate high-performance electrochemical biosensors for the detection of mycotoxins.

## 1. Introduction

As one of the secondary metabolites produced by Aspergillus flavus and parasiticus, aflatoxin B1 (AFB_1_) is classified as a Group I carcinogen by the World Health Organization’s Cancer Research Institute [1,2,3,4]. The tolerance of AFB_1_ in food should be less than 20 ppb, as reported by the Food and Agriculture Organization of the United Nations [5,6]. Due to its dramatic poisoning effects on the human body, the rapid and accurate detection of AFB_1_ is in high demand in food processing [7,8]. So far, various analytical methods such as high-performance liquid chromatography, thin-layer chromatography and enzyme-linked immunosorbent assays have been developed to detect AFB_1_ [9,10,11,12,13]. However, most of these methods involve costly equipment and complicated sample processing and suffer from several drawbacks such as a low efficiency and/or false-positive results, which greatly restrict their widespread use [14,15,16]. Alternatively, electrochemical sensors have drawn increasing attention in detecting mycotoxins, owing to their high efficiency and simplicity [15,17,18,19]. For instance, Shi et al. developed a novel electrochemical immunosensor for AFB_1_ detection based on Au nanoparticles supported by graphene, which showed a limit of detection (LOD) of 0.001 ng mL^−1^ [20]. In addition, Wang et al. synthesized a reusable electrochemical sensor to detect AFB_1_ in white grape wine and milk. The resulting sensor exhibited a LOD of 0.0019 ng mL^−1^ [19].

Aptamers (Apt) are a key component of electrochemical biosensors [21,22,23]. The specific sequences of Apt with different spatial structures play a central role in recognizing AFB_1_ [24]. Goud et al. reported the performances of two Apt sequences of AFB_1_ (seqA and seqB) modified with aminos that had LODs of 0.12 ng mL^−1^ and 0.25 ng mL^−1^, respectively [25]. Chen et al. designed an aptasensor for AFB_1_ detection using an amino-terminal and thiol-terminal AFB_1_-complementary Apt [26]. The proposed aptasensor exhibited a good performance with a LOD of 0.0036 ng mL^−1^. Jia et al. developed a fluorescent aptasensor with a TAMRA Apt [27]. The aptasensor showed a good performance with a LOD of 0.35 ng mL^−1^. The Apt used in our previous study had a high affinity and selectivity for AFB_1_ [28]. In this work, the 5′ and 3′ ends of the same Apt modified with -SH and -NH_2_, respectively, were used for the aptasensor fabrication. The structures of the electrode also greatly affect the performance of the resulting electrochemical sensors. Self-supporting electrodes (SSEs) are widely used in electrocatalysis [29]. The three-dimensional structures of SSEs not only provide a large surface area, but also afford the fast transport of reactants and products [30]. In addition, the portability of SSEs also offers a prominent advantage for the practical applications of the resulting electrochemical sensors [31]. Carbon-based materials are ideal substrates for building SSEs [32,33]. For instance, Zhe et al. fabricated a self-supporting binder-free electrode using carbon cloth for the detection of nitrite, resulting in a LOD of 0.04 µM [34]. In addition, a carbon nanotube (CNT) is also a promising substrate for the development of superminiaturized chemical and biological sensors due to its high sensitivity to electronic properties and unparalleled large unit surface [35,36,37,38,39]. Geng et al. developed an efficient SSE using Ni/MoC encapsulated by nitrogen-doped carbon nanotube arrays on carbon cloth for overall water splitting [29].

In this work, a high-performance SSE was fabricated using a hierarchical carbon nanofiber/carbon felt (CNF/CF) monolith, which was prepared via a chemical vapor deposition process. To the best of the authors’ knowledge, the production of the same electrode material for AFB_1_ detection has not been reported so far. We further constructed an electrochemical biosensor for the quantitative detection of AFB_1_ on the basis of oxidation of hydrogen peroxide (H_2_O_2_) catalyzed by HRP. The developed biosensor generated a remarkable performance for AFB_1_ detection, owing to the unique structures.

## 2. Materials and Methods

### 2.1. Chemicals

1-Hydroxy-2,5-pyrrolidinedione (NHS), 4-(2-hydroxyethyl)-1-piperazineethanesulfonic acid (HEPES) and N-Ethyl-N′-(3-dimethylaminopropyl) carbodiimide hydrochloride (EDC) were purchased from Sigma-Aldrich, Shanghai, China. Ochratoxin A (OTA, 10 µg mL^−1^ in acetonitrile) was purchased from Pribolab Bioengineering Co., Ltd. (Qingdao, China). AFB_1_ (≥98.0%), bovine serum albumin (BSA, 20 mg mL^−1^) and horseradish peroxidase (HRP, activity ≥250 U mg^−1^) were obtained from Sangon Biotech (Shanghai, China). Potassium ferricyanide (K_3_[Fe(CN)_6_]), oxalic acid, nickel (II) nitrate hexahydrate, magnesium chloride (MgCl_2_), copper (II) nitrate trihydrate, glucose (G), citric acid (CA), starch, ascorbic acid (AA), potassium chloride (KCl), sodium chloride (NaCl), chloroauric acid (HAuCl_4_), sodium hydroxide (NaOH), disodium phosphate (Na_2_HPO_4_), methanol, potassium dihydrogen phosphate (KH_2_PO_4_) and H_2_O_2_ with a mass concentration of 30% were purchased from Congyuan Instrument Co., Ltd. (Guangzhou, China). Sulfhydryl- and amino-modified Apts of AFB_1_ with a sequence of (5′-SH-modified) 5′-TGG GGT TTT GGT GGC GGG TGG TGT ACG GGC GAG GG-3′ (3′-NH_2_-modified) were obtained from Aiji Biotechnology Co., Ltd. (Guangzhou, China). All reagents were used as received without further purification. A 0.01 M phosphate buffer (PBS) consisting of 8 g of NaCl, 0.2 g of KCl, 1.44 g of Na_2_HPO_4_ and 0.24 g of KH_2_PO_4_ in an aqueous solution was used; the pH was adjusted to 7.4 by adding NaOH.

### 2.2. Preparation of the SSE

The SSE was prepared according to the method reported in our previous work [40]. First, the CF (3.0 × 3.0 × 0.5 cm) was thermally activated at 420 °C for 10 h using a tube furnace under an air atmosphere. A total of 1.11 g of oxalic acid was dissolved in 30 mL of ethanol and 1.7 g of nickel (II) nitrate hexahydrate and 0.7 g of copper (II) nitrate trihydrate was dissolved in 30 mL of ethanol. The metal precursor solution was titrated with the oxalic acid solution under magnetic stirring. Subsequently, the mixture was heated in an autoclave at 120 °C for 8 h and cooled to 25 °C afterward. The sample was washed with deionized water three times and then dried at 80 °C for 8 h. The powder sample was ultrasonically dispersed in ethanol to form a suspension with a concentration of 5 mg mL^−1^. The activated CF was repeatedly immersed in the Ni-Cu oxalate suspension until the load was about 320~390 mg. The CF loaded with Ni-Cu oxalates was calcined at 600 °C for 2 h with a heating rate of 10 °C min^−1^ under a methane and nitrogen flow (volumetric ratio: 3:1).

### 2.3. Synthesis of the Gold-Modified SSE (Au/SSE)

The Au/SSE was prepared via a displacement reaction. The details of the experimental procedures were as follows: three pieces of SSE (1.5 × 0.6 × 0.25 cm) were immersed in 0.5 mM of HAuCl_4_ at 50 °C for 24 h. After the reaction, the SSE was washed thoroughly with deionized water and then dried at 50 °C for 8 h.

### 2.4. Preparation of the BSA-Apt-Au/SSE

For the synthesis of Apt-Au/SSE, the PBS containing 1 µM Apt was introduced dropwise to the Au/SSE. After reacting for 10 min, the electrode was washed with deionized water to remove the residual solution. After that, 5 mg mL^−1^ of the BSA solution was added dropwise to the Apt-Au/SSE to passivate the non-specific active sites that were not bound to the Apt. The as-prepared BSA-Apt-Au/SSE was then washed three times and dried for further use.

### 2.5. Preparation of the Spiked Samples

A total of 5 g of wine and soy sauce was added to a methanol solution with a concentration of 0.2 g mL^−1^. The mixed solution was ultrasonically dispersed and then centrifuged at 10,000 rpm for 10 min. The supernatant was then obtained for the preparation of the AFB_1_ spiked samples. A solution of methanol and chloroform (*v*/*v* = 1:1) was used to dissolve AFB_1_.

### 2.6. Biosensor Fabrication

**Scheme 1:**
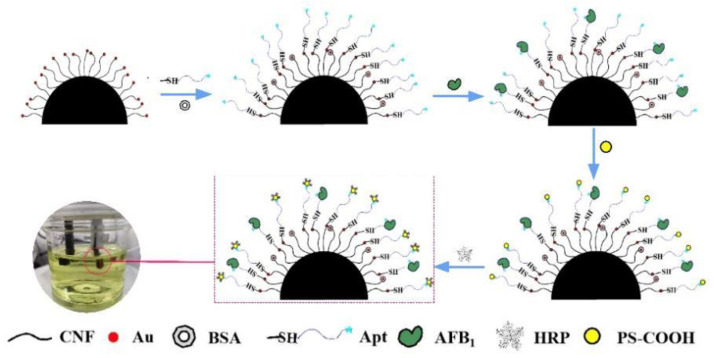
Stepwise illustration of fabrication procedure of the electrochemical biosensor based on the self-supported electrode.

Carboxylated polystyrene (PS-COOH) nanospheres were prepared according to our previous work [28]. Before the biosensor fabrication, the PS-COOH was activated using a carboxyl activation solution that contained 20 mM of HEPES (pH = 6.8), 5 mM of EDC and 5 mM of NHS. The BSA-Apt-Au/SSE electrodes were immersed in the AFB_1_ solution at room temperature for 2 h. The obtained electrode was denoted as AFB_1_-BSA-Apt-Au/SSE. The AFB_1_-BSA-Apt-Au/SSE was then immersed in the PS-COOH suspension for 30 min, thoroughly washed with deionized water and dried at room temperature. Finally, the modified electrode, denoted as PS-BSA-Apt-Au/SSE, was immediately immersed in 2 mg mL^−1^ of HRP at 4 °C for 30 min. The prepared electrodes are shown in Figure S1. The biosensor preparation process is shown in Scheme 1.

### 2.7. Structural Characterization and Electrochemical Analysis

For the electrochemical analysis, a CHI760E electrochemical workstation was used. The electrochemical measurements were carried out on a conventional three-electrode system. The biosensor, carbon rod and Ag/AgCl were used as the working, counter and reference electrodes, respectively. PBS containing 10 mM of H_2_O_2_ was used as the electrolyte for the differential pulse voltammetry (DPV) measurements. The DPV curves were recorded at potentials ranging from −0.7 to −0.2 V vs. Ag/AgCl with a scan rate of 1 mV s^−1^. The peak current (I_p_) of H_2_O_2_ electro-oxidation was recorded. Amperometric i-t curves were recorded at a potential of −0.35 V. The PBS containing the 10 mM H_2_O_2_ solution was used as the electrolyte. Cyclic voltammetry (CV) curves were recorded at potentials of −0.2 ~ +0.6 V with a scan rate of 10 mV s^−1^. All the electrochemical measurements were conducted at an ambient temperature and pressure. All the potentials reported in this work were referenced to Ag/AgCl. The morphology of the samples was observed by field emission electron scanning microscopy (FESEM).

## 3. Results

### 3.1. Structural and Electrochemical Characterization

**Figure 1:**
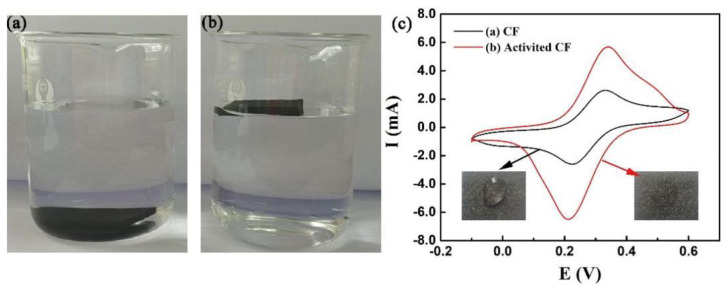
Digital photos of the hydrophilicity of the CF materials before and after thermal treatment. (**a**) Activated CF; (**b**) pristine CF. (**c**) CVs of the CF (black line) and activated CF (red line) were recorded in 1 mM [Fe(CN)_6_]^3^^−^^/4^^−^ with a scan rate of 10 mV s^−^^1^.

Before the electrode fabrication, the pristine CF was activated via a thermal treatment to improve its hydrophilicity. The improved hydrophilicity of the CF after the thermal treatment could be verified from the hydrophilic experiment shown in Figure 1a,b. The water droplet could quickly permeate into the activated CF whereas it remained on the surface of the pristine CF. In addition, the activated CF could quickly sink to the bottom whereas the pristine CF floated on the water. The CVs of the CF before and after the thermal treatment are shown in Figure 1c. The activated CF showed an enhanced current intensity compared with that of the pristine CF, which may have been due to the better hydrophilicity of the activated CF.

**Figure 2:**
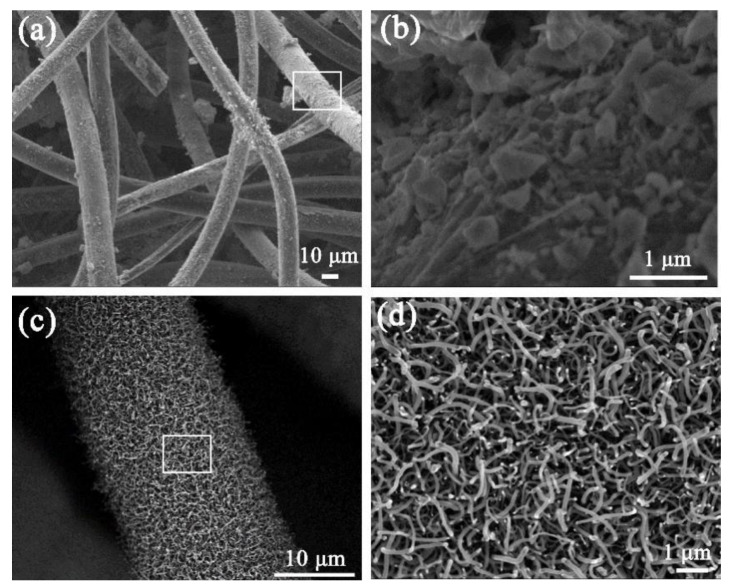
FESEM micrographs of Ni-Cu oxalate-decorated CF (**a**,**b**) and SSE (**c**,**d**) at different scales.

Figure S2a,b are the FESEM micrographs of the pristine CF, showing that the pristine CF was of a porous structure consisting of intricately interlacing carbon fibers. The diameter of the carbon fiber was about 10 µm. Figure 2a,b are the images of the CF loaded with Ni-Cu oxalates. It could be seen that the Ni-Cu oxalates were evenly distributed on the CF. Figure S3 and Figure 2a,b show that the SSE electrode maintained a well-defined hierarchical structure after the in situ growth of the carbon nanofibers. After the catalytic methane decomposition reaction, many dense carbon nanotubes were present on the surface of the CF. Notably, the Ni-Cu nanoparticles were deposited on the tip of the nanotubes. The macropores of the original CF still existed, regardless of the growth of the carbon nanofibers. The FESEM image of the as-prepared PS-COOH is shown in Figure S3b. The PS-COOH showed a uniform spherical structure with an average diameter of ca. 100 nm.

**Figure 3:**
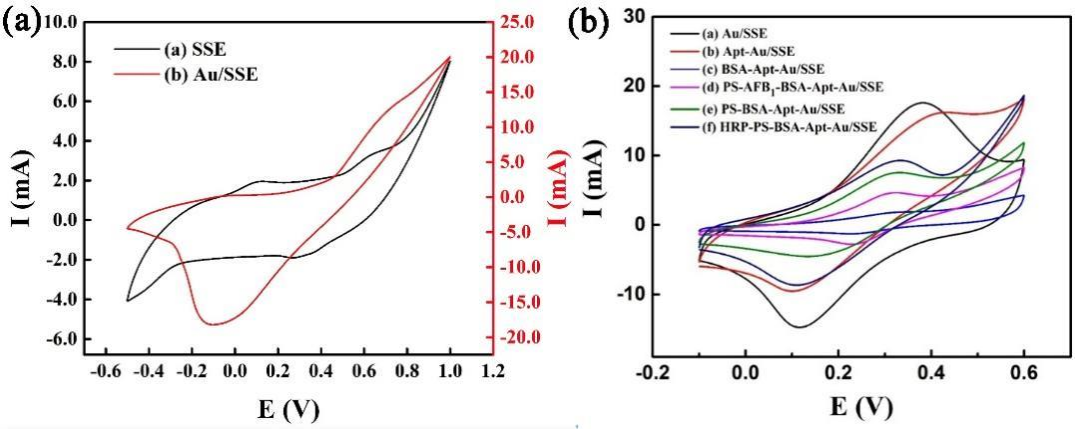
(**a**) CVs of the SSE (black line) and Au/SSE (red line) electrodes recorded in 0.1 M NaOH with a scan rate of 10 mV s^−^^1^. (**b**) CVs of the as-prepared electrodes recorded in 1 mM [Fe(CN)_6_]^3−^ with a scan rate of 10 mV s^−^^1^.

The electrodes before and after the replacement reaction were characterized by CV in the 0.1 M NaOH electrolyte; the results are shown in Figure 3a. For the SSE electrode, the characteristic peaks at +0.1 V and +0.63 V may have been related to the oxidation of Cu and Ni, respectively [41,42,43,44]. For the Au/SSE electrode, the peaks related to Ni and Cu oxidation became less distinctive because Au partially replaced the Ni/Cu atoms on the surface of the nanoparticles. The peaks at +0.7 V and −0.1 V could be ascribed to the Au redox, which suggested that Au had been successfully introduced into the electrode [45,46].

### 3.2. Fabrication of the Electrochemical Biosensor

Scheme 1 illustrates the procedures for the fabrication of the electrochemical biosensor. The electrochemical biosensor was fabricated based on the Au/SSE electrode. The Au/SSE electrode had a porous structure with a large surface area, which served as an ideal platform for the electrochemical sensors, owing to the facile substance transfer. In addition, it had a three-dimensional structure that could improve the performance of the biosensor. The fabrication procedures of the electrochemical biosensor were as follows. First, Au atoms were introduced into the Ni-Cu nanoparticles by replacement reactions. The functionalized Apt was then immobilized on the electrode through the Au-S bonds. Before the AFB_1_ addition, the non-specific active sites were passivated by BSA. The Apt on the electrode specifically bound to AFB_1_ through van der Waals forces, the hydrogen bond and/or hydrophobic forces. The free Apt bound to the activated PS-COOH through the amido bond. As there were many activated carboxyl groups in the PS-COOH, HRP with amino groups could combine with them through amido bonds. Meanwhile, the iron porphyrin ring in the HRP catalyzed the redox of H_2_O_2_ in the electrolyte, which served as the basis for the quantitative detection of AFB_1_ via the electrochemical signal of H_2_O_2_.

### 3.3. Electrochemical Results of the Electrochemical Biosensors

Figure 3b shows the CVs of the as-prepared electrodes in the 1 mM K_3_[Fe(CN)_6_] electrolyte. The Au/SSE exhibited two characteristic peaks at +0.11 and +0.38 V, which were related to the redox of the [Fe(CN)_6_]^3−/4−^ [47,48,49]. After Apt binding, the I_p_ value decreased and the peak potential positively shifted, which could be ascribed to the fact that the Apt as a bio-macromolecule does not have electrochemically active sites. This result also confirmed that the Apt was successfully assembled on the electrode. After passivating the non-specific active sites on the electrode surface with BSA, most of the active sites of the electrode material were covered by BSA and Apt, thus leading to a minimum value of the I_p_. After the BSA-Apt-Au/SSE electrode was modified by the PS-COOH, the I_p_ value increased due to the electrochemically active sites on the PS-COOH. However, the I_p_ value of the PS-BSA-Apt-Au/SSE electrode decreased after the AFB_1_ addition due to the combination of AFB_1_ and Apt, which gave rise to fewer PS-COOH nanospheres on the electrodes. The steric hindrance of AFB_1_ may also have hindered the charge transfer in the electrolyte. When the PS-BSA-Apt-Au/SSE electrode was modified by HRP, a relatively higher I_p_ value was noted, indicating that the HRP was successfully immobilized on the electrode. In addition, the integration of the PS-COOH with HRP, serving as a signal amplification probe, greatly enhanced the current of the electrode.

### 3.4. Detection of AFB_1_

**Figure 4:**
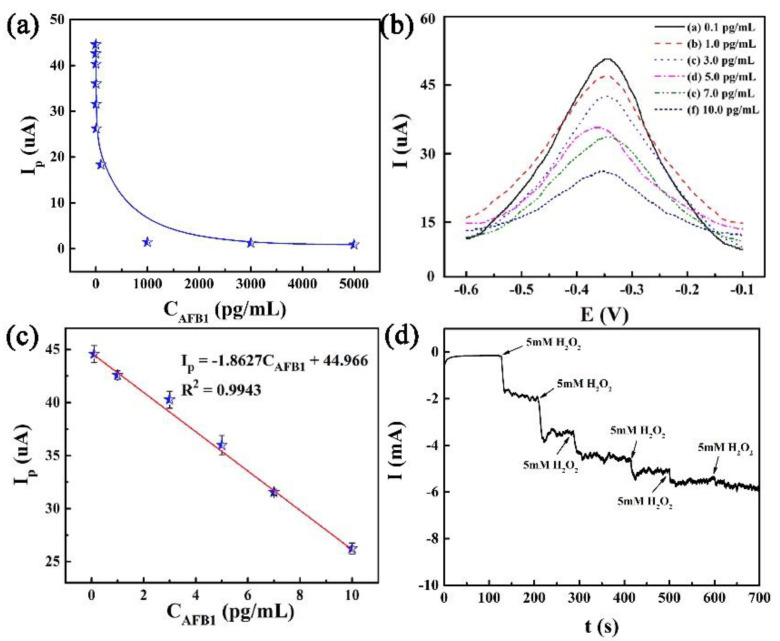
(**a**) I_p_ response of the biosensor to different concentrations of AFB_1_ in a large concentration range of 0~5000 pg mL^−^^1^. DPV responses of the biosensor recorded in 10 mM H_2_O_2_ with varying concentrations of AFB_1_ (**b**) and corresponding calibration curves of the biosensor for the detection of AFB_1_ (**c**). Error bars were obtained from three independent measurements. (**d**) I-t curve of the biosensor recorded in varying H_2_O_2_ concentrations.

The relationship between the I_p_ and the concentration of AFB_1_ is shown in Figure 4a. It indicated that the biosensors exhibited distinct responses to different concentrations of AFB_1_ because of the varying amounts of HRP immobilized on the electrode. It was found that there was a linear relationships between the I_p_ and the concentrations of AFB_1_ in the low concentration ranges. In all cases, the I_p_ gradually decreased with an increasing concentration of AFB_1_. Figure S4 and Figure 4b are the DPV curves of AFB_1_ within the high and low concentration ranges, respectively. Figure 4c demonstrates that the I_p_ was inversely proportional to the AFB_1_ concentration from 0.1 to 10 pg mL^−1^ with a square of the correlation coefficient R^2^ of 0.9943. The linear relationship could be described as I_p_ = −1.8627 C_AFB1_ + 44.966, where C_AFB1_ denotes the concentration of AFB_1_. According to the method reported by Shrivastava and Gupta, the calculation formula of the LOD is LOD = 3L/b, where 3L is the standard deviation based on three times the blank sample measurement and b is the slope of the calibration curve [50]. The LOD of the electrochemical biosensor was 0.016 pg mL^−1^. For three independent electrodes, the relative standard deviations (RSDs) of the I_p_ values at different concentrations of AFB_1_ were less than 3%. The LOD of the biosensor was closely related to the three-dimensional structure of the self-supporting electrodes. The results showed that the prepared biosensors were successfully applied to the quantitative analysis of AFB_1_.

### 3.5. Effects of H_2_O_2_ Concentration

To evaluate the effects of the H_2_O_2_ concentration on the electrolyte, i-t was applied. Figure 4d shows the electrochemical response of the electrochemical biosensor with different concentrations of H_2_O_2_. For each time, 5 mM of H_2_O_2_ was added to the electrolyte and the current change was observed. It could be seen that the current gradually increased with an increasing H_2_O_2_ concentration. However, the magnitude of the increase became smaller after 10 mM of H_2_O_2_ was added. Therefore, 10 mM of H_2_O_2_ was considered to be the optimal concentration in the electrolyte solution for the detection of AFB_1_.

### 3.6. Stability, Reproducibility and Specificity

**Figure 5:**
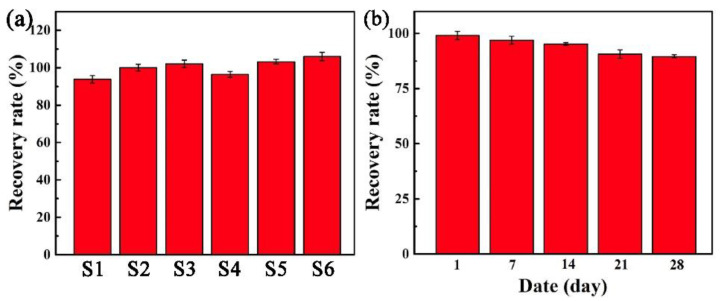
(**a**) Reproducibility of six electrode samples (S1–S6). (**b**) Stability of an as-prepared electrode. Error bars shown were obtained from three independent measurements.

For the practicability of an electrochemical biosensor, the stability, reproducibility and specificity of the biosensor are important. To evaluate the reproducibility of the biosensors, six electrode samples (S1–S6) were prepared at the same time. The test was repeated three times on each electrode and the RSD was calculated. Figure 5a shows the results of the reproducibility. The recovery rates were in the range of 93.8~105.9%. In addition, the RSDs of the recovery rate values from each electrode of the six samples were less than 3%. The results indicated that the developed electrochemical biosensors had a good reproducibility. For the stability of the biosensors, the prepared BSA-Apt-Au/SSE electrodes were prepared and stored in a sealed container at 4 °C for four weeks. They were used to detect 10 pg mL^−^^1^ of AFB_1_ per week. The corresponding results are shown in Figure 5b. The current responses slightly decreased with the increasing storage time. After four weeks, the recovery rates of the electrochemical biosensors reduced to 89.6%. The excellent stability of the biosensor could be attributed to the three-dimensional structure of the SSE and the robustness of the Apt.

**Figure 6:**
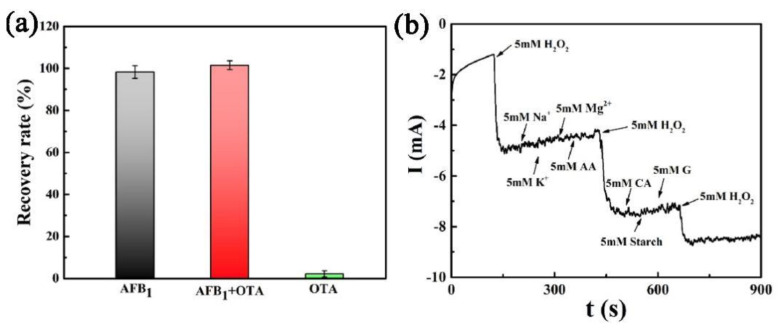
(**a**) Recovery rates of the developed biosensors for the detection of AFB_1_ and the mixture of AFB_1_ and OTA. Error bars were obtained from three independent measurements. (**b**) Selectivity test of the biosensor toward H_2_O_2_ by interference with Na^+^, K^+^, Mg^2+^, AA, CA, starch and G.

As a few impurities may have existed in the actual detection process, different toxins, ions and macromolecular organics were used as interference to test the specificity of the biosensors. Figure 6 shows the results of the interfering toxin OTA, ions and macromolecules on the electrode. When only OTA (1 pg mL^−^^1^) was present, the recovery rate was 2.1%, which may have been due to the absorption of the PS-COOH in/on the electrode via non-chemical forces. When both AFB_1_ (1 pg mL^−1^) and OTA (1 pg mL^−1^) were present, the recovery rate was similar to that of AFB_1_ (1 pg mL^−1^) alone. In addition, tests of other interferential substances were performed by i-t. The details are as follows. The same concentration (5 mM) of the interfering substances and H_2_O_2_ were added to the electrolyte at regular time intervals (60 s). It was found that the current did not change after adding the interfering substances whereas it significantly increased after adding H_2_O_2_. Therefore, the results confirmed that the electrode material could specifically recognize AFB_1_ without being interfered with by OTA, Na^+^, K^+^, Mg^2+^, AA, CA, starch and G.

### 3.7. Real Sample Analyses

Due to the portability of materials, electrochemical biosensors based on self-supporting materials have unique advantages in applications. To verify the operability of the electrochemical biosensor in actual samples, the concentrations of AFB_1_ in different linear ranges of two real samples of wine and soy sauce were detected. A known amount of AFB_1_ (1 pg mL^−^^1^, 5 pg mL^−^^1^, 10 pg mL^−^^1^, 1 ng mL^−^^1^, 3 ng mL^−^^1^ and 5 ng mL^−^^1^) was added to the two samples. Table 1 and Table 2 show the recovery rates of the low and high concentration ranges of AFB_1_ in wine and soy sauce, respectively. The recovery rates of the low and high concentration ranges of AFB_1_ in wine were between 95.5~106.7% and 89.0~101.3%, respectively. In soy sauce, the recovery rates were between 94.1~101.6% and 87.5~92.3%, respectively. The RSDs of all detections were less than 2%. It showed that the biosensors had an outstanding accuracy, reliability and operability. These results indicated that the developed biosensor could be used to detect AFB_1_ in complex samples. To further highlight the advantages of the electrochemical biosensors prepared in this work, a comparison of the performance of the biosensor with that of other electrochemical sensors reported in the literature is shown in Table 3. The existing methods for AFB_1_ detection have many limitations such as a complicated operation, low accuracy, high detection cost and long detection time. Despite the complicated preparation process of the preliminary electrodes, the detection accuracy of ABF_1_ was high and the detection time was relatively short. To be consistent, the performance tests were immediately conducted once the electrodes had been prepared.

## 4. Conclusions

In summary, a free-standing electrochemical biosensor with multi-scaled nanostructures was manufactured for the detection of AFB_1_ on the basis of oxidation of H_2_O_2_ catalyzed by HRP. A three-dimensional CNF/CF composite was utilized as a platform for the fabrication of the electrochemical sensors. Au was introduced into the electrode via a replacement reaction with Ni-Cu nanoparticles and further employed as a binder with Apt whilst the PS-COOH nanospheres were assembled into the support to afford abundant sites for immobilizing the HRP, thereby amplifying the electrochemical signals. The electrochemical results indicated that the current signal was closely related to the concentration of AFB_1_. The linear calibration ranges of AFB_1_ were 0.1~10 pg mL^−1^ and the LOD was 0.016 pg mL^−1^. The recovery rates of the detection of AFB_1_ in wine and soy sauce were between 87.5% and 106.7%, respectively. The resulting electrochemical biosensors possessed prominent advantages of outstanding sensitivity and stability in the detection of AFB_1_, owing to the synergistic effects of the components. The free-standing feature of the electrochemical sensor afforded a facile operation during practical applications. The fabrication strategy reported in this work could be extended to prepare other electrochemical sensors by varying the Apts of target substrates.

## Figures and Tables

**Table 1:** Recovery of low-concentration AFB_1_ in wine and soy sauce for the application of biosensors in a real matrix.

Real Sample	Spiked AFB_1_ (pg mL^−1^)	Found AFB_1_ (pg mL^−1^)	Recovery (%)	RSD * (%)
Wine	1.00	0.954	95.5	0.84
	5.00	4.782	95.6	1.09
	10.0	10.671	106.7	1.88
Soy sauce	1.00	0.941	94.1	0.20
	5.00	5.082	101.6	1.27
	10.0	10.042	100.4	1.62

* RSD values were determined from their parallel experiments.

**Table 2:** Recovery of high-concentration AFB_1_ in wine and soy sauce for the application of biosensors in a real matrix.

Real Sample	Spiked AFB_1_ (ng mL^−1^)	Found AFB_1_ (ng mL^−1^)	Recovery (%)	RSD (%)
Wine	1.00	0.890	89.0	0.42
	3.00	2.837	94.6	0.29
	5.00	5.065	101.3	1.04
Soy sauce	1.00	0.922	92.3	1.36
	3.00	2.746	91.5	0.26
	5.00	4.376	87.5	0.96

**Table 3:** Comparison of electrochemical sensors reported in the literature for the detection of aflatoxin AFB_1_.

Sample	Recovery (%)	LOD (pg mL^−1^)	Biosensor Type	Electrode Type	Detection Mode	Reference
Wine	89.0~106.7	0.016	Aptasensor	Au/SSE	Catalyzing the decomposition of hydrogen peroxide	Present work
Soy sauce	87.5~101.6	30				
Corn	95.6~103.5	4.3	Aptasensor	Gold electrode	Coupling of DNA walker with hybridization chain reaction	[51]
Wheat	94.0~102.2					
Maize	95.0~102.0	31.2	Electrical optical biosensor	Gold nanobipyramids/indium tin oxide	Immobilization of anti-AFB_1_ antibodies	[52]
Wheat	85.9~111.6	27	Immunosensor	Gold nanoparticles/carbon nanofibers	Specific binding with AFB_1_	[49]
Grain	92.2~109.5	50	Immunosensor	Gold electrode	Direct competitive enzyme-linked immunoassay	[53]

## Data Availability

Not applicable.

## Supplementary Materials

The following supporting information can be downloaded at: https://www.mdpi.com/article/10.3390/bios12100897/s1, Figure S1: The picture of the as-prepared BSA-Apt-Au/SSE electrode. Figure S2: FESEM micrographs of the pristine CF in different scales (a) 100 µm, (b) 10 µm. Figure S3: FESEM micrographs of (a) SSE and (b) PS-COOH. Figure S4: DPV responses of the biosensor recorded in 10 mM H_2_O_2_ with varying concentrations of AFB_1_.
