# Supplementary material for: A High-Performance Self-Supporting Electrochemical Biosensor to Detect Aflatoxin B1
Source: Biosensors (Basel). 2022 Oct 20;12(10):897. doi: 10.3390/bios12100897 (PMC9599888; doi:10.3390/bios12100897)
Supplement: Supplementary file 1 [file biosensors-12-00897-s001.zip › biosensors-1933246-supplementary.pdf]

Supporting Information for

A high-performance self-supporting electrochemical biosensor to detect aflatoxin B1

Yunfei Zhang,[1+] Tingting Lin,[1+], Yi Shen,[*1,2,3] and Hongying Li[4]

[1]School of Food Science and Engineering, South China University of Technology, Guangzhou, 510640, China.

[2]Overseas Expertise Introduction Center for Discipline Innovation of Food Nutrition and Human Health (111 Center) Guangzhou, 510640, China.

[3]China-Singapore International Joint Research Institute, Guangzhou Knowledge City, Guangzhou 510663, China.

[4] Institute of High-Performance Computing, Agency for Science, Technology and Research, 138632, Singapore.

*Correspondence: Yi Shen (feyshen@scut.edu.cn)

[+] The authors contributed equally to this work.

Reagents

1-Hydroxy-2,5-pyrrolidinedione (NHS), 4-(2-hydroxyethyl)-1-piperazineethanesulfonic acid (HEPES) and N-Ethyl-N'-(3-dimethylaminopropyl) carbodiimide hydrochloride (EDC) were purchased from the Sigma-Aldrich, China. Ochratoxin A (OTA, 10 ug mL^{-1} in acetonitrile) was purchased from the Pribolab Bioengineering Co., Ltd. AFB_1 (⩾ 98.0%), bovine serum albumin (BSA, 20 mg mL^{-1}) and horseradish peroxidase (HRP, activity ⩾ 250 U mg^{-1}) were obtained from the Sangon Biotech (Shanghai, China). Potassium ferricyanide ($K_3[Fe(CN)_6]$), oxalic acid, nickel (II) nitrate hexahydrate, magnesium chloride ($MgCl_2$), copper (II) nitrate trihydrate, glucose (G), citric acid (CA), starch, ascorbic acid (AA), potassium chloride (KCl), sodium chloride (NaCl), Chloroauric acid ($HAuCl_4$), Sodium hydroxide (NaOH), disodium phosphate (Na_2HPO_4), methanol, potassium dihydrogen phosphate (KH_2PO_4) and H_2O_2 with a mass concentration of 30% were purchased from the Congyuan Instrument Co., Ltd. (Guangzhou, China). Sulfhydryl- and amino- modified Apt of AFB_1 with a sequence of (5'-SH modified) 5'-TGG GGT TTT GGT GGC GGG TGG TGT ACG GGC GAG GG-3' (3'-NH_2 modified) were obtained from the Aiji Biotechnology Co., Ltd. (Guangzhou, China). All reagents were used as received without further purification. 0.01 M phosphate buffer (PBS) consists of 8 g of NaCl, 0.2 g of KCl, 1.44 g of Na_2HPO_4 and 0.24 g of KH_2PO_4 in an aqueous solution and the pH was adjusted to 7.4 by adding NaOH.

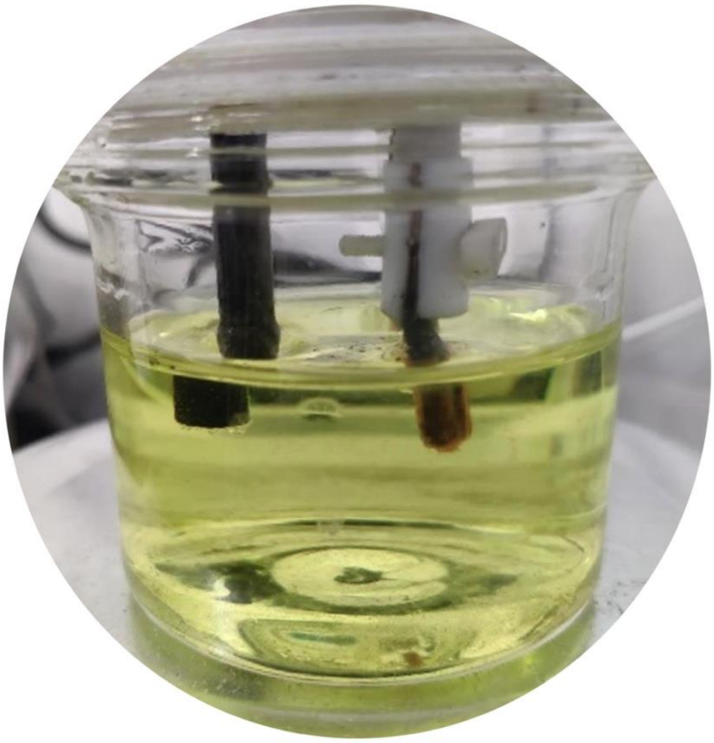

Figure S1. The picture of the as-prepared BSA-Apt-Au/SSE electrode.

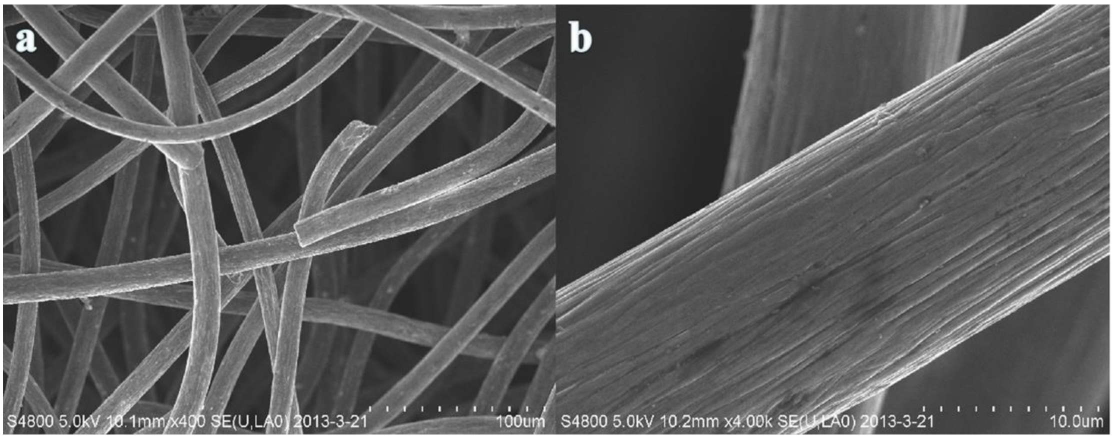

Figure S2. FESEM micrographs of the pristine CF in different scales (a) 100 µm, (b) 10 µm.

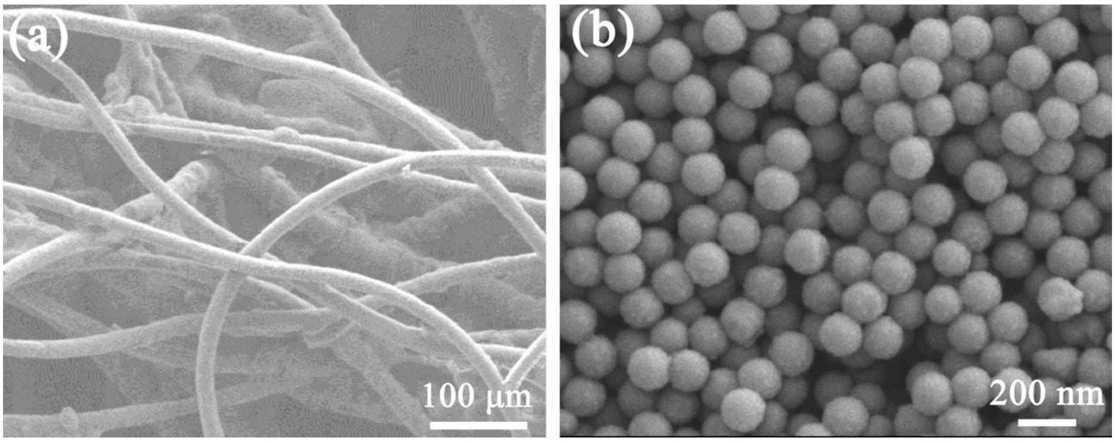

Figure S3. FESEM micrographs of (a) SSE and (b) PS-COOH.

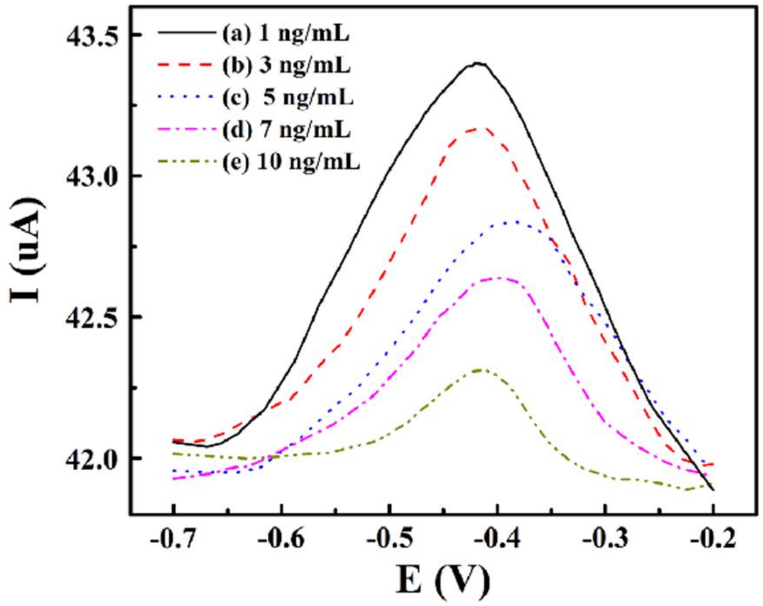

Figure S4. DPV responses of the biosensor recorded in 10 mM H_2O_2 with varying concentrations of AFB_1.
